# Obesity and Exercise: New Insights and Perspectives

**DOI:** 10.1210/endrev/bnaf017

**Published:** 2025-06-16

**Authors:** Natasha Maria James, Kristin I Stanford

**Affiliations:** Dorothy M. Davis Heart and Lung Research Institute, The Ohio State University Wexner Medical Center, Columbus, OH 43210, USA; Department of Surgery, Division of General and Gastrointestinal Surgery, The Ohio State University Wexner Medical Center, Columbus, OH 43210, USA; Dorothy M. Davis Heart and Lung Research Institute, The Ohio State University Wexner Medical Center, Columbus, OH 43210, USA; Department of Surgery, Division of General and Gastrointestinal Surgery, The Ohio State University Wexner Medical Center, Columbus, OH 43210, USA

**Keywords:** obesity, metabolic adaptations, exercise modulations, molecular mechanisms, combinatorial therapies

## Abstract

Obesity is increasing rapidly worldwide and is projected to affect approximately half the US population by the year 2035. Obesity is a complex condition, and individuals who have obesity are at greater risk for developing associated metabolic diseases such as type 2 diabetes (T2D), metabolic dysfunction–associated steatohepatitis (MASH), and cardiovascular diseases (CVD). Understanding the underlying factors which contribute to obesity and that impact key molecular mechanisms of metabolic organs such as adipose tissue, liver, and muscle is crucial for combating the disease. Exercise is a well-established measure to prevent or mitigate the adverse consequences of obesity, with several beneficial effects to whole-body metabolism and adaptations to metabolic tissues. This review explores the impact of obesity on the development of metabolic diseases. Specifically, we will discuss: how obesity alters metabolic function and the potential benefits of exercise; the specific effects of obesity and exercise on muscle, adipose tissue, and liver; and potential effects of pharmacotherapeutics or bariatric surgery in combination with exercise.

## Essential Points Covered in the Review

- Obesity affects multiple metabolic pathways in metabolic organs, specifically white adipose tissue, brown adipose tissue, liver, and skeletal muscle
- Exercise studies provide compelling evidence to influence obesity-induced alterations in metabolic organs
- The potential of exercise, in combination with bariatric surgery and incretin therapy, presents a promising area for future research targeted at advancing therapeutic strategies to combat obesity

Obesity is drastically increasing, with the World Health Organization (WHO) indicating a 2-fold increase in the prevalence of obesity from 1990 to 2022, and an estimation of approximately half the US population having obesity by 2030 (1). The increasing presence of obesity correlates with the increased risk of cardiovascular disease (CVD), metabolic dysfunction–associated steatohepatitis (MASH), and type 2 diabetes mellitus (T2D), among others, highlighting the critical importance of understanding mechanisms and strategies to combat obesity (2-4).

Exercise is a compelling therapeutic tool to combat obesity and metabolic disease (5-9). Among the various forms of exercises, aerobic and resistance training are often investigated in terms of their role to induce molecular adaptations to key metabolic organs such as adipose tissue, liver, and skeletal muscle (5, 7, 8, 10-16). Recent strategies to combat obesity, including bariatric surgery and weight loss drugs, have gained increasing popularity in promoting weight loss with improvements in overall health including cardiovascular outcomes and glucose homeostasis (17-19). The latest emerging research studies have investigated the potentially synergistic combination of exercise and weight loss drugs or bariatric surgery (20, 21).

**Figure 1.**
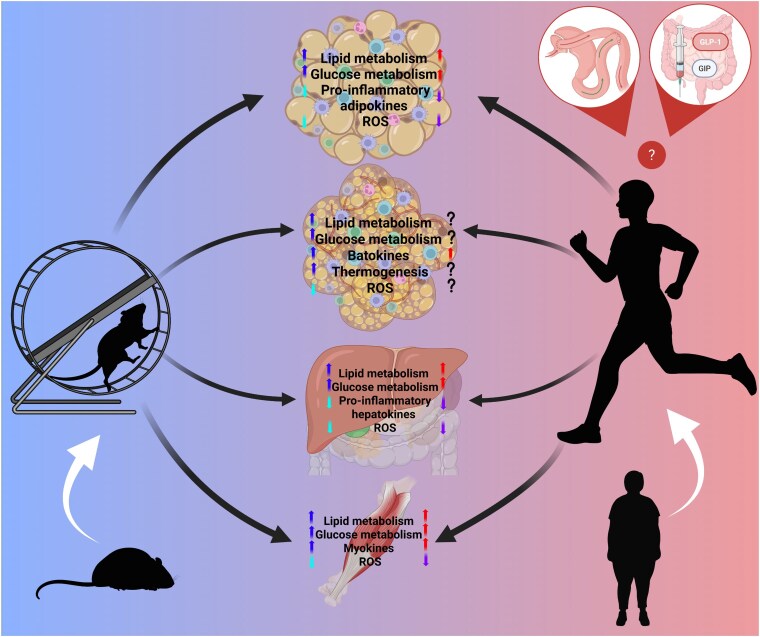
Exercise modulates potent beneficial effects to various metabolic organs impacted by obesity such as increasing (upward arrow) lipid and glucose metabolism and reducing (downward arrow) inflammation for white and brown adipose tissue, the liver, and skeletal muscle in humans and animals. Alternative therapies, including bariatric surgery and incretin therapy, provide a unique perspective of possible combinatorial interventions to attenuate the undesirable effects of obesity. Figure made in Biorender.

In this review, we will discuss the effects of obesity on various metabolic organs including adipose tissue, liver, and skeletal muscle, in both animal models and human studies. We will explore how obesity impacts various metabolic processes, including mitochondrial function, thermogenic capacity, endocrine regulation, and glucose and lipid metabolism, as well as how exercise influences these outcomes in the context of obesity. Finally, we discuss weight loss interventions, such as incretin therapies and bariatric surgery, and their potential effectiveness in combination with exercise and the consideration of multiple factors, such as potential compensatory lifestyle changes and ensuring inclusive courses of treatment when addressing obesity and its possible therapeutic strategies (Fig. 1).

### Obesity and the Potential Benefits of Exercise

Obesity is increasing at epidemic proportions across the United States and worldwide, and the rise in obesity is concomitant with an increase in several obesity-related diseases. The most common obesity-associated diseases include T2D, CVD, and MASH (22, 23). T2D is a chronic metabolic disease characterized by high blood glucose levels and impaired insulin homeostasis (24, 25). CVD includes diseases that affect the circulatory system of the body, such as the heart and vasculature (26). Increased fat accumulation in the liver in conjunction with inflammation and fibrosis results in MASH (27). The risk of other diseases, including cancer and Alzheimer's Disease, are also increased in people with obesity (28, 29), highlighting the significance of obesity as a comorbidity.

Exercise is an important therapeutic tool to combat obesity and obesity-related disorders (30-32). Exercise is a well-established tool to improve aerobic capacity, resting heart rate, blood pressure, and overall metabolic health (33-35). Additionally, exercise can mediate indices such as body mass index (BMI), waist circumference (WC), hip circumference, body fat percentage (BFP), insulin resistance, and waist to height ratio, which have been shown to be significant risk factors in determining metabolic health and associated diseases (10, 36-42). Among multiple different forms of exercise, aerobic exercise and resistance training are the most well-studied with regard to impacting and potentially improving anthropometric measures in individuals with obesity (5, 7-9). Exercise is also known to affect key molecular pathways adversely impacted by obesity, including mitochondrial activity and glucose and lipid metabolism in adipose tissue, liver, and skeletal muscle (43-46).

While exercise improves metabolic health and upregulates multiple metabolic pathways, individuals with obesity-associated metabolic diseases such as T2D have reduced expression of genes involved in mitochondrial biogenesis and oxidative phosphorylation in muscle (47, 48). Mitochondrial DNA (mtDNA) and oxidative phosphorylation are also diminished in white adipose tissue (WAT), correlating with increased adipose tissue inflammation and insulin resistance (49). Studies have demonstrated that in people with T2D, aerobic exercise increases whole-body insulin sensitivity by ∼20% and reduces HbA1C levels by 0.8% (45, 50, 51), while high-intensity interval training (HIIT) and moderate intensity continuous training (MICT) elevates expression of genes involved in muscle mitochondrial activity and lipid utilization (45). In patients with MASH, moderate exercise decreases hepatic triglyceride content and circulating free fatty acids, enhances glucose and insulin sensitivity, and reduces pro-inflammatory cytokines such as IL-6 and TNF-α (31, 52). Similarly, physical activity of any level or intensity reduces the risk factors for CVD including BMI, fasting glucose, and systolic blood pressure (32, 53 , 54). The reduction in CVD risk factors regardless of intensity of physical activity is important because measurements used to assess physical activity and the effects of exercise can vary in people with obesity. For example, maximal oxygen consumption (VO₂_max_), which is a key indicator of aerobic fitness, can be interpreted as absolute VO₂_max_, reflecting intrinsic aerobic capacity, or can be adjusted for fat-free mass or lean body mass, offering a more accurate measure of muscle endurance in obese individuals (55).

### Effects of Obesity and Exercise on Metabolic Tissues

Exercise attenuates the effects of obesity by inducing molecular adaptations to distinct organs. Crucial metabolic organs impacted by obesity include adipose tissue, liver, and skeletal muscle. Specific exercise-induced adaptations to these metabolic tissues that can combat obesity are discussed below.

#### Exercise and white adipose tissue in obesity

Adipose tissue is a highly dynamic tissue that adapts to changes in energy demand. White adipose tissue (WAT) is primarily responsible for insulation and energy storage. It consists of white adipocytes alongside various other cell types (56, 57). WAT is divided into 2 main types: subcutaneous adipose tissue (scWAT) and visceral adipose tissue (vWAT). Both store lipids as triglycerides, which can then be mobilized and used for energy (58). Subcutaneous WAT is found beneath the skin and is linked to better insulin sensitivity and glucose regulation (59, 60). In contrast, visceral WAT surrounds abdominal organs and is associated with insulin resistance (61). These 2 depots differ in their adaptations to exercise and associations with insulin sensitivity, suggesting distinct physiological functions of these 2 subclasses of WAT.

**Figure 2.**
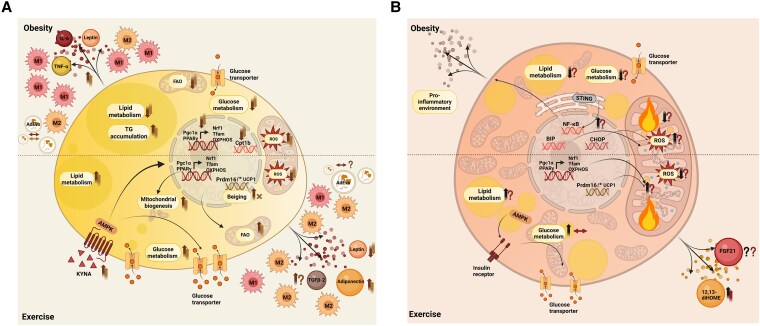
a) Molecular alterations to white adipose tissue (WAT) in conditions of obesity and the effect of exercise on altered mechanisms in animals and humans. Obese conditions lead to a pro-inflammatory state of WAT with an increase in M1 macrophages, pro-inflammatory adipokine release, enhanced triglyceride (TG) accumulation and reactive oxygen species (ROS) generation. Lipid and glucose metabolic pathways are downregulated and mitochondrial activity is reduced such as fatty acid oxidation. Exercise potentially mitigates these adverse effects through various modulations, specifically an increase in transcription of mitochondrial activity genes such as Nrf1 and Tfam, a decrease in ROS generation, increase in beiging marker Prdm16 (specific to animals), increased glucose uptake via glucose transporter translocation to the membrane and release of anti-inflammatory adipokines and AdEVs. Arrows indicate alterations reported in animals (dark brown; arrows on left) and humans (light brown; arrows or ´x´on right). b) Molecular alterations to brown adipose tissue (BAT) in conditions of obesity and the effect of exercise on altered mechanisms in animals and humans. Obese conditions lead to a pro-inflammatory state of BAT, accumulation of ROS generation and downregulation of lipid and glucose metabolic pathways. Exercise has profound effects on the various obesity-induced modulations, specifically an increase in transcription of mitochondrial activity genes such as Nrf1 and Tfam, a decrease in ROS generation, increase in beiging marker Prdm16 (specific to animals), increased glucose uptake via glucose transporter translocation to the membrane and release of batokines. Arrows indicate alterations reported in animals (black) and humans (red). Figure made in Biorender. Abbreviations: AdEVs; adipose-derived extracellular vesicles; AMPK; AMP-activated protein kinase; BIP, binding immunoglobulin protein; CHOP, C/EBP homologous protein; CPT1B; carnitine palmitoyltransferase 1B; KYNA; kynurenic acid; NF-κB, Nuclear factor kappa B; Nrf1; nuclear respiratory factor 1; OXPHOS; oxidative phosphorylation; PGC1α, peroxisome proliferator-activated receptor γ coactivator 1 α; PPARγ, peroxisome proliferator-activated receptor γ; PRDM16, PR domain containing 16; Tfam; mitochondrial transcription factor A; UCP1, uncoupling protein 1.

Exercise-induced adaptations to WAT include an increase in mitochondrial activity and endocrine function in humans (62-65) and enhanced thermogenic gene expression alongside mitochondrial activity in rodents (13, 66-71). Exercise also induces sex-specific adaptations in humans (63, 72) and rodents (70, 71, 73), demonstrating the importance of investigating both sexes to completely understand the exercise-induced effects on WAT. In this section, we will discuss obesity-associated alterations to WAT, specifically inflammation, mitochondrial activity, endocrine activity and thermogenic remodeling, and how exercise affects these modulations (Fig. 2A, Table 1).

**Table 1.** Key findings on the effects of exercise on WAT and BAT in potentially mitigating obesity-induced changes to adipose tissue in animal and human studies

WAT	Intervention	Outcomes
Animal Studies	11 days of voluntary wheel cage training in mice (13)	Increased expression of beige adipocyte markers such as UCP1 and Prdm16 and presence of multilocular cells in scWAT of trained mice Increased basal OCR of scWAT from trained mice Increased vascularization markers such as Vegfa, pdgf in scWAT of trained mice
	2 hours of daily swimming for 4 weeks in rats (74)	Increased pgc1α expression in vWAT and scWAT of mice
	11 days of voluntary wheel cage training in mice (67)	Increased expression of mitochondrial function markers such as pgc1α, nrf1, tfam, and UCP1 in scWAT of trained mice Increased basal OCR and maximal respiratory capacity of scWAT from trained mice Increased basal OCR of vWAT from trained mice
	6 weeks of training with either voluntary wheel cage training or treadmill training in HFD mice (66)	Both training modalities increased Cd137 expression (beiging marker) increased citate synthase activity in scWAT of trained mice No changes in scWAT mitochondrial respiratory capacity with either of the training modalities was observed in trained mice
	8 weeks of training, 5 days/week, 45 minutes/day of aerobic (treadmill training) or resistance training (ladder climbing with weights) or HIIT (treadmill training at varying speeds) in obese mice (75)	Increased expression of pg1α and UCP1 in vWAT of aerobic exercise-trained mice
	1 month of a swimming protocol for 90 minutes daily, 5 days/week in mice (76)	Increased expression of pgc1α, nrf1, tfam, UCP1, and COX IV was observed in scWAT of wild type trained mice.
	15 weeks of HIIT or moderate intensity exercise via treadmill training for 5 days/week in mice in addition to 12.5% calorie restriction in obese mice (77)	No changes in thermogenic markers such as UCP1, Prdm16, Dio2, and Fgf21 in scWAT and vWAT of both training groups Decrease in UCP1 expression in vWAT of HIIT-trained mice
	8 weeks of treadmill training, 45 minutes/day, 5 days/week in HFD mice (78)	Decreased expression of mitochondrial protein in scWAT of trained mice
	6 weeks of voluntary wheel cage training exercise in obese mice (79)	Decreased expression of TNF-α, MCP-1, PAI-1 and IKKβ in vWAT of obese trained mice Decreased plasma leptin levels of obese trained mice
	12 weeks of resistance training (ladder climbing with weights) 3 days/week and aerobic training for up to 60 minutes/day, 5 days/week via treadmill in diabetic rats. The rats de-trained for 4 weeks post exercise intervention (69)	Decreased circulating TG, LDL-C, leptin, TNF-α, and fasting blood glucose levels in resistance and aerobic trained diabetic rats Increased circulating insulin levels in resistance and aerobic trained diabetic rats De-training resulted in an increase in body weight and circulating TG, leptin and TNF-α levels in both exercise-trained diabetic rats
	8 weeks of treadmill training, 5 days/week up to 60 minutes in HF/HS mice (80)	Improved glucose tolerance in HF/HS trained mice No changes in circulating adiponectin levels in HF/HS trained mice
	11 days of voluntary wheel cage training in HFD mice (68)	Increased circulating levels of Tgfβ2 in trained chow diet and HFD mice Increased mRNA expression of Tgfβ2 in scWAT and vWAT and increased protein expression of Tgfβ2 in scWAT of trained chow fed mice.
	11 days of voluntary wheel cage in mice (81)	Increased expression of Rilpl2 and Myo5a in scWAT of trained mice
	4 weeks of voluntary wheel cage running in HFD mice (82)	Increased expression of circadian rhythm genes including Dbp, Tef, Nr1d2, and Per3 in scWAT and vWAT of trained mice Decreased expression of ECM remodeling genes thbs1 and sparc in scWAT and vWAT of trained mice
Human Studies	3 weeks of exercise training consisting of 30-60 minutes of interval training and 50 minutes of aerobic training. Training sessions were alternated between the 2 protocols each day. The study groups included previously active and sedentary individuals (62)	Pg1α and cpt1β expression and mtDNA content were significantly higher in scWAT of active individuals before training. Training did not affect the expression of UCP1, Prdm16, pgc1α, and cpt1β mRNA levels in scWAT of both groups. No effect on expressions of beige-specific genes such as CD137 and TBX1 of both groups.
	2 weeks consisting of 6 sessions of sprint interval and MICT up to 60 minutes in IR and healthy participants (83)	Exercise training increased glucose uptake and decreased fatty acid uptake in scWAT and vWAT in both IR and healthy groups. Enhanced adipose tissue vasculature and decreased CD36 and ANGPTL4 expression in scWAT of IR exercised individuals.
	8 weeks of strength and aerobic exercises, 3 times/week in women with obesity (63)	Increased citrate synthase activity in scWAT of exercised cohort Decreased mitochondrial uncoupled respiration and UCP1 expression in exercised cohort.
	6 weeks of aerobic training, 4 sessions/week of up to 40 minutes in overweight men (84)	Exercise did not affect mRNA expression of brown and beiging markers such as UCP1 and CD137 in scWAT of trained individuals.
	12 weeks of plyometric exercise combined with HIIT, 3 days/week in females with obesity (64)	Reduced plasma leptin concentration and leptin/adiponectin ratio in HIIT + plyometric group Reduced plasma HOMA-IR in HIIT + plyometric group
	6 weeks of jump rope exercise training, 5 days/week for 40 minutes/day in males with obesity (65)	Increased adiponectin levels in exercised group
	7 months of endurance training, 4-5 days/week, 30-60 minutes/day in females with obesity (85)	Decreased circulating leptin and TNFα levels and increased adiponectin levels in exercised group
	Acute bout of exercise for 30 minutes in individuals with T2D (86)	Increased expression of oncostatin-M in scWAT post exercise

BAT	Intervention	Outcomes
Animal Studies	Exercise training via treadmill, 1 hour/day, 6 times each week for 4 weeks (87)	Upregulation of the insulin/AMPK signaling pathway and PPAR/VEGF signaling pathway in BAT of exercised mice Downregulation of the Jak-STAT/ErbB/TGF-beta signaling pathway in BAT from exercised mice Upregulated VEGF and COX2 pathway in BAT from exercised mice
	Exercise via treadmill training for 40 minutes/day, 5 days/week for 8 weeks in HFD mice (88)	Increased brown adipocyte progenitor cells from exercised mice Increased differentiation of brown pre-adipocytes into brown adipocytes and UCP1 expression in vitro from exercised mice
	Exercise via treadmill training for up to 20 minutes and up to 5days/week for 12 months in obese female mice (89)	Increased expression of pgc1a, prdm16 and UCP1 in BAT from exercised mice
	Exercise via swimming for 1 hour/day, 5 days/week for 6 weeks in HFD-induced metabolic syndrome rats (90)	Increased expression of UCP1 and PPARϒ-2 in BAT of exercised mice
	Acute bout of exercise via treadmill for 40 minutes and chronic exercise via voluntary wheel cage running for 3 weeks (91)	Increased circulating levels of batokine 12,13-diHOME
Human Studies	6 sessions of MICT or HITT in 2 weeks, up to 60 minutes of each session in healthy men (92)	Exercise training decreased insulin-stimulated glucose uptake in BAT of individuals with high BAT activity Exercise training had no effect on insulin-stimulated glucose uptake in BAT of individuals with low BAT activity
	24 weeks of endurance and resistance training, 3-4 times/week (150 minutes/week of endurance training and 80 minutes/week of endurance training) in healthy adults (93)	No changes in glucose uptake level in BAT in the exercise trained participants

Abbreviations: 12,13-diHOME, 12,13-dihydroxy-9Z-octadecenoic acid; AMPK, AMP-activated protein kinase; BAT, brown adipose tissue; BFP, body fat percentage; ECM, extracellular matrix; HFD, high-fat diet; HF/HS, high-fat/high-sugar; HIIT, high-intensity interval training; IL-, interleukin; IR, insulin resistant; LDL-C, low-density lipoprotein cholesterol; MICT, moderate intensity continuous training; OCR, oxygen consumption rate; scWAT, subcutaneous white adipose tissue; T2D, type 2 diabetes; TG, triglyceride; TNF-α, tumor necrosis factor alpha; Ucp1, uncoupling protein 1; vWAT, visceral white adipose tissue; WAT, white adipose tissue.

##### Inflammation in WAT

Obesity induces various adaptations to WAT (94-96). Adipocytes undergo both an increase in size (hypertrophy) and number (hyperplasia) to accommodate increased fat storage in obesity (97). The increase in fat storage disrupts multiple cellular mechanisms, including mitochondrial biogenesis and glucose and lipid metabolism, all of which have detrimental effects on the normal function of adipocytes (43, 49, 98, 99). Obesity is associated with low-grade inflammation of WAT and infiltration of pro-inflammatory M1 macrophages and increased tumor necrosis factor alpha (TNF-α) and interleukin 6 (IL-6) expression (100, 101). Additionally, WAT releases pro-inflammatory adipokines such as TNF-*α*, IL-6, leptin, and resistin, which promotes inflammation of WAT (102). The obesity-associated inflammation and increased free fatty acids contribute to adipose tissue insulin resistance (94).

In contrast, exercise decreases obesity-associated inflammation and reduces fibrosis in WAT and improves glucose and insulin homeostasis (12, 103, 104). Six weeks of wheel cage exercise in mice with diet-induced obesity (DIO) reduces expression of inflammatory markers such as TNF-α in the vWAT, while 12 weeks of aerobic and resistance training reduces circulating levels of TNF-α in mice with high-fat diet (HFD)-induced glucose intolerance (69, 79). An important pathway that mediates inflammation in WAT in response to exercise is the kynurenine pathway (105, 106). The kynurenine pathway is a catabolic pathway that breaks down tryptophan to generate an intermediate metabolite kynurenine (KYN) which can be further processed into kynurenic acid (KYNA) with the oxidized form of nicotinamide adenine dinucleotide (NAD+) as the final product (107). In mice, increased circulating KYN impairs insulin sensitivity and lipid homeostasis in adipocytes through the aryl hydrocarbon receptor (AhR)/signal transducer and activator of transcription 3 (stat3)/IL-6 signaling pathway, suggesting the impact of excess KYN accumulation adversely affecting metabolic health (108). Recent studies have shown that circulating KYNA, the metabolically beneficial byproduct of KYN metabolism, is significantly increased with exercise in mice (109). The increase in KYNA reduced palmitate-induced inflammation and insulin resistance in adipose tissue and skeletal muscle of HFD mice via the G protein–coupled receptor 35 (Gpr35)/AMP-activated protein kinase (AMPK) and elevated sirtuin 6 (SIRT6) pathways (110). Treatment with KYNA increased AMPK phosphorylation, elevated SIRT6 expression, promoted fatty acid oxidation in muscle, and inhibited fat storage in adipose tissue, while inhibition of AMPK and SIRT6 via siRNA results in the reversal of KYNA-mediated lipogenesis in 3T3-L1 adipocytes and fatty acid oxidation gene expression in C2C12 myocytes. In addition, 2 weeks of KYNA treatment improved glucose tolerance and reduced weight gain in mice fed HFD (111). Mechanistically, KYNA activates GPR35, leading to the upregulation of thermogenic genes such as peroxisome proliferator-activated receptor-γ coactivator 1-α (Pgc1α), PR domain containing 16 (Prdm16), and cell death inducing DFFA like effector A (CIDEA), and expression of oxidative phosphorylation (OXPHOS) in WAT (111). These findings highlight KYNA's potential role in maintaining systemic metabolic balance (111).

Studies in humans have shown that exercise training is associated with a reduction of adiposity, BMI, BFP, and circulating inflammatory cytokines such as IL-6 and TNF-α (112, 113). A recent study has shown that 3 weeks of aerobic training resulted in adaptations to the scWAT of overweight women, with a significant decrease in levels of transcripts and proteins related to inflammation and extracellular matrix without an impact on body and fat mass, suggesting molecular adaptations to mechanisms of WAT rather than a direct reduction of WAT mass (114). Aerobic and resistance training programs up to 4 months resulted in a decrease in scWAT inflammatory gene expression such as IL-6, IL-8, and TNF-α and CD36 macrophage marker in patients with obesity or those over 71 years of age (115, 116). Similar to animal models, elevated levels of KYN are associated with a higher BMI in humans (117), while plasma KYNA levels are increased up to 63% in active males participating in endurance training 1 hour post exercise (118).

In summary, in mice, exercise reduces obesity-associated inflammation and fibrosis in WAT, improves glucose and insulin homeostasis, and increases KYNA levels, which enhance fatty acid oxidation, reduce fat storage, and improve glucose tolerance. Similarly, in humans, exercise reduces adiposity, BMI, % body fat, and inflammatory cytokines such as IL-6 and TNF-α and increases KYNA levels.

##### Exercise-induced regulation of mitochondrial activity in WAT

In adipocytes, as in other cells, mitochondria govern crucial mechanisms such as regulating glucose and lipid homeostasis and ATP production through OXPHOS (119-122). Obesity is associated with mitochondrial dysfunction in adipocytes; this can be attributed to the fact that under obese conditions there is a substrate overload due to increased lipid and glucose availability, resulting in amplified OXPHOS and consequently an increase in reactive oxygen species (ROS) as a byproduct of the OXPHOS cycle (123-126).

In rodent models fed HFD, the mitochondrial function of adipocytes is severely impaired (123-126); mitochondrial proteins such as PGC1α are decreased, and there is an increase in ROS and mitochondrial fragmentation via fission, ultimately resulting in mitophagy (123-126) Additionally, mice with an adipose tissue–specific PGC1α deletion, when challenged with a HFD, develop insulin resistance and a reduction in OXPHOS proteins in the WAT, further emphasizing the pivotal role of mitochondria in adipocytes to maintain metabolic homeostasis (127).

In other rodent studies, a 12-week HFD resulted in a significant reduction in genes and metabolites associated with mitochondrial glucose oxidation, including 1,5-anhydroglycol (1,5-AG), a plasma marker of short-term glycemic regulation. Additionally, glucose-6-phosphate, a key glycolytic intermediate, was reduced, alongside decreased pyruvate dehydrogenase lipoamide kinase isozyme 4 (Pdk4) expression, which plays a role in suppressing mitochondrial pyruvate dehydrogenase activity (98). With regard to lipid oxidation, studies in rats with DIO have shown reduced fat oxidation and lower carnitine palmitoyl transferase I (CPT1) mRNA expression in vWAT, suggesting impaired mitochondrial fatty acid oxidation (99). Similar to in vivo studies, in vitro studies in 3T3-L1 cells have shown that exposure to high glucose and free fatty acid induces morphological changes to the mitochondria, an increase in the mitofission protein DRP1, and a decrease in mitochondrial biogenesis proteins PGC1α and nuclear respiratory factor 1 (NRF1) (128).

Exercise, however, significantly enhances mitochondrial function in rodents (13, 66, 67, 74, 75). Four weeks of swimming exercise increased expression of Pgc1a in vWAT and scWAT (74). In fact, our lab has shown that as little as 11 days of voluntary wheel cage running upregulates expression of several genes involved in mitochondrial activity such as PGC1α, Nrf1, mitochondrial transcription factor A (Tfam) and uncoupling protein 1 (UCP1) in scWAT (67). These increases in gene expression were correlated to improved functional outcomes, as adipocytes differentiated from the stromal vascular fraction from vWAT and scWAT of exercised mice had increased basal oxygen consumption rates and maximal respiratory capacity when compared to cells isolated from sedentary mice (67). Exercise has been reported to affect mitochondrial function in WAT under conditions of obesity. Eight weeks of treadmill training increased Pgc1α and UCP1 expression in vWAT (75), while 6 weeks of treadmill exercise increased citrate synthase activity in scWAT of HFD-trained mice (66).

In humans with obesity, multiple transcription factors of mitochondrial biogenesis in WAT, including PGC1α, NRF1, TFAM, and OXPHOS proteins, are significantly reduced (43, 44, 129). In fact, individuals with obesity have an increase in circulating oxidative stress markers, including plasma thiobarbituric acid reactive substance (TBARS) and urinary 8-epi-prostaglandin-F2α (8-epi-PGF2α), both of which correlate with high BMI and increased WC (123). Mitochondrial oxygen consumption rates and citrate synthase specific activity are also significantly decreased in WAT of individuals with obesity, and this negatively correlates with BMI and body weight (BW) (130, 131). Interestingly, studies examining WAT of monozygotic twin pairs who are lean or obese have revealed a decrease in mitochondrial DNA and PGC1α and OXPHOS protein, correlating an increase in genes associated with inflammatory pathways and thus suggesting a decrease in mitochondrial function as a response to obesity (43 , 49, 132).

In humans, moderate to vigorous aerobic exercise for 3 weeks increased mitochondrial DNA content and expression of adipose regulatory genes peroxisome proliferator-activated receptor gamma (PPARγ) and Cpt1β in scWAT, in healthy subjects (62), but these increases were not seen in healthy male or female subjects after 6 weeks of HIIT (133).

In contrast to rodent studies, the exercise-induced mitochondrial adaptations in individuals with obesity is complex. Twelve weeks of combined aerobic and resistance training increases mitochondrial respiration of scWAT via enhanced expression of complex I and II of the electron transport chain in women with moderate obesity and a BMI of 30 to 40 kg/m^2^ (134). Moreover, 8 weeks of combined aerobic and strength training increased mitochondrial energy production in scWAT, and elevated citrate synthase activity in women with obesity (63). Interestingly, 12 weeks of combined aerobic and resistance exercise did not affect OXPHOS and mitochondrial biogenesis markers in the scWAT of men with obesity (72). A further understanding of how various exercise modalities, exercise duration, and sex influence modifications to WAT depots, specifically the mitochondria, in the context of obesity warrants further investigation.

##### Thermogenic remodeling of WAT

An important exercise-induced adaptation to rodent adipose tissue is a “beiging” of scWAT (67, 135). Adipose tissue is a highly plastic tissue, and the plasticity of white adipocytes is bi-directional; cold stress and exercise induce a “beige” phenotype to increase thermogenic capacity, while obesity does the opposite and increases a “whitening” of the adipose tissue in animal models (136-138).

In rodents, exercise induces a beige phenotype in scWAT which is sustained up to 3 weeks post-exercise training (13, 67, 76, 135). This phenotype is observed more prominently in male rodents, as well as an increase in genes and pathways related to lipid utilization, aerobic metabolic pathways, tissue remodeling, and angiogenesis, while exercise in female rodents enhances pathways involved in adipogenesis and insulin signaling (70, 73).

In rodent models of HFD, the role of exercise to induce beiging has been inconsistent; studies have reported differing effects of exercise on the expression of Ucp1, a mitochondrial protein which facilities non-shivering thermogenesis, dissipating energy in the form of heat (139, 140). One study demonstrated that 8 weeks of aerobic exercise increased expression of UCP1 in scWAT of HFD mice but decreased mitochondrial content protein (74), while others showed that 15 weeks of HIIT or moderate intensity exercise had no effect on thermogenic markers including UCP1 and Prdm16 in scWAT of HFD mice (77, 78). These studies shed light on the fact that alterations to UCP1 gene expression are not a direct measure of its activity and subsequent metabolic outcomes, and the findings indicate that to uncover the functional relevance of an increase in UCP1, other direct measures of thermogenic capacity such as indirect calorimetry and infrared thermography are essential.

Interestingly, exercise does not induce a beiging of scWAT in humans. Multiple studies have reported no difference in the expression of beiging markers UCP1 and Prdm16 in lean and obese populations after exercise (72, 84, 141). In contrast, studies have shown that the tissue does have the capability to beige, but exercise is not an effective stimulus (142, 143). Several hypotheses have been brought forward to address the phenomenon of the exercise-induced beiging observed in rodents. Firstly, in contrast to cold and pharmacological stimuli, which trigger an increase in thermogenesis to compensate for heat loss, exercise itself is a heat-generating activity (144-146). Another interesting perspective is that exercise decreases the size of lipid droplets and overall adipocyte size in scWAT, leading to reduced insulation and a potential cold stress, warranting the need for increased thermogenesis in rodents (147-149). In line with this idea is the fact that at room temperature, which for humans is ∼20-22 °C, mice are under a minor cold stress (150). The optimal comparable temperature for mice to study metabolic responses is thermoneutral conditions, which is 30 °C (150). Importantly, when mice are exercised at thermoneutral temperatures, the beiging effect of WAT is blunted, supporting the idea that cold stress contributes to the beiging of scWAT in rodents (151, 152).

Collectively, these findings suggest that exercise triggers the thermogenic remodeling of WAT in rodents and does not induce beiging in human scWAT. Future studies focused on addressing these differences, with a focus on obesity, could potentially provide greater insight and translational relevance to the exercise-induced adaptations of WAT.

##### Endocrine function of WAT

WAT secretes a myriad of adipokines which play a crucial role in regulating energy storage and expenditure, glucose and lipid metabolism, inflammatory responses, and insulin sensitivity (68, 153-157). Adipokines can act in an autocrine, paracrine, or endocrine manner. Here we will discuss the endocrine function of WAT, specifically leptin, adiponectin, and transforming growth factor beta receptor 2 (TGF-β2), oncostatin-M and adipose-derived extracellular vesicles (AdEVs).

###### Leptin

Leptin is produced by adipose tissue and plays an essential role in maintaining the balance between energy intake and energy expenditure by binding to leptin receptors (LepR) (153). LepR is expressed on several organs throughout the body, including the hypothalamus of the brain (158-160). Leptin binding to LepR results in the activation of downstream pathways and subsequently increased energy expenditure and reduced food intake (153).

In animals, circulating leptin levels are proportional to body fat mass, with increasing obesity leading to increased leptin concentrations (154). In mice, treatment with leptin reduces hyperglycemia and improves insulin resistance (161-164). Leptin activates AMPK in other metabolic tissues, which promotes fatty acid oxidation, reduces fat accumulation, and enhances insulin sensitivity (165). Chronic exercise lowers BW, which coincides with reduced leptin levels in both obese and nonobese conditions (166, 167).

In humans, individuals with obesity have higher leptin levels compared to lean individuals (155). Elevated leptin is also associated with an increased incidence of metabolic syndrome (168, 169). While short-term and moderate intensity exercise do not significantly impact leptin levels, chronic training (up to 12 months) reduces circulating leptin, and this is associated with a decrease in % body fat and fat mass (170-173). An additional study found that 12 weeks of HIIT combined with plyometric training reduced leptin levels, which coincided with reduced fat mass and with improvements in lean body mass in obese subjects (64).

###### Adiponectin

Adiponectin is one of the most abundant adipokines, predominantly expressed in WAT, and known for its anti-inflammatory, anti-obesity, and antidiabetic properties (156, 157). In animal models of obesity and diabetes, administration of adiponectin improves hyperglycemia, while its absence leads to reduced insulin sensitivity (174-176). Exercise elevates plasma adiponectin in rodents, alleviating metabolic disorders such as T2D and CVD (80, 177-179). Furthermore, 10- to 12-week exercise interventions have been associated with increased expression of adiponectin-related myocardial receptors in apolipoprotein E protein knockout mice and improved endothelial function in the aorta of T2D mice (178, 179).

Similarly in humans, adiponectin levels are reduced in individuals with obesity and diabetes across all age groups, whereas increased adiponectin is linked to improved insulin resistance (180-186). Genetic mutations in adiponectin-related genes are associated with a heightened susceptibility to metabolic disorders (187-189). Human studies indicate that exercise training, ranging from 6 weeks to 24 months, results in increased adiponectin levels, coinciding with improved triglyceride levels, insulin sensitivity, and cardiorespiratory fitness which correlated with a decrease in body fat (65, 85, 190). Notably, just 2 or 3 sessions of aerobic exercise can elevate adiponectin levels by 260% independent of changes in BW. Additionally, physical training enhances the expression of adiponectin receptors in muscle, as well as AMPK, highlighting adiponectin's potential role in mediating insulin resistance in individuals with metabolic syndrome (191, 192).

###### Transforming growth factor beta receptor 2

To evaluate whether exercise-induced adaptations to WAT contribute to beneficial effects on metabolic health, our lab investigated the effects of transplantation of scWAT from exercise-trained mice into sedentary mice (13). Interestingly, scWAT transplanted from exercise-trained mice resulted in improved glucose tolerance of recipient mice at 9 days post-transplantation even under HFD conditions (13, 68). The exercise-trained scWAT mediated metabolic improvements via the adipokine TGF-β2, which is involved in fatty acid and glucose metabolism (68). Additionally, mice with an adipose tissue–specific deletion of TGF-β2 did not display exercise-induced systemic glucose uptake, emphasizing its crucial role in metabolic adaptations (68).

In humans, 12 weeks of endurance exercise increase expression of TGF-β2 in the scWAT of healthy male subjects (68) and a combination of MICT, HIIT and resistance training for 6 weeks and a 2-week high-intensity training protocol increased circulating TGF-β2 levels in healthy male subjects (68). Together these data identified a previously unknown role for exercise-induced adipokine TGF-β2 to regulate glucose and lipid metabolic pathways that may affect metabolic health.

###### Adipose tissue extracellular vesicles

Extracellular vesicles (EVs) are a diverse group of lipid-enclosed nanoparticles that function as messengers between tissues when released into the extracellular space (193-195).The main categories of EVs include exosomes, microvesicles, and apoptotic bodies (196). Various cell types, including adipocytes, secrete EVs (AdEVs), which contain bioactive molecules such as microRNAs (miRNAs), messenger RNAs (mRNAs), DNA, proteins, lipids, and metabolites. These EVs are believed to play a significant role in obesity and its associated comorbidities (197-200).

An important study highlighted the critical role of circulating AdEVs; in mice lacking the adipose-specific miRNA-processing enzyme Dicer, circulating miRNA levels were significantly reduced. Transplantation of adipose tissue reversed this effect, underscoring the importance of adipose tissue as a source of circulating miRNAs (201). Rodent studies have shown that AdEVs contribute to obesity through various cargoes. For example, a previous study demonstrated that treatment with vWAT-EVs from obese mice induced insulin resistance in recipient mice (202). This effect was mediated by retinol binding protein 4 (RBP4) in vWAT-EVs, which activated macrophages and promoted an inflammatory state by increasing IL-6 and TNF-α production. Similarly, other studies have shown that vWAT-EVs from obese mice have reduced levels of miR-141-3p, a miRNA involved in AKT phosphorylation in recipient hepatocytes, which enhances insulin signaling. A decrease in miR-141-3p levels impaired insulin signaling in vitro, leading to reduced insulin sensitivity (203).

Aerobic exercise in DIO mice alters circulating miRNA levels, which correlate with miRNA expression in both the liver and WAT. Specifically, miR-22 levels were negatively correlated with the expression of adipogenesis and insulin sensitivity markers in WAT, as well as the presence of liver steatosis (204).

In individuals with obesity, changes to the size, number, and cargo composition of AdEVs have been reported, with implications for insulin signaling and inflammatory pathways (205-208). Importantly, several miRNAs, including miR-23b, miR-4429, miR-148b, and miR-4269, are differentially expressed in adipocytes from lean and obese individuals. Pathway analysis revealed alterations in TGF-β and Wnt/β-catenin signaling, suggesting an impact on the development and progression of inflammatory and fibrotic activities (209).

Studies examining the effects of exercise on EVs in humans have shown that acute bouts of exercise rapidly increase EV release into circulation, with signatures linked to endothelial cells and leukocytes in healthy male subjects (210-212). Interestingly, when comparing normal-weight and male and female subjects with obesity, it was found that normal-weight individuals exhibited higher levels of microvesicles after exercise than individuals with obesity. Additionally, exercise reduced circulating EVs more in male than female individuals (213). While these studies highlight the role of EVs in obesity and exercise, they primarily focus on circulating EVs. Further research specifically targeting AdEVs in response to obesity and exercise is needed to better understand these changes and their potential for therapeutic exploitation in metabolic diseases. These data show that exercise has profound effects on WAT, including changes in endocrine activity, and identify a unique role for adipose tissue–mediated EV communication as a potential contributor to improved metabolic health.

###### Exercise induces adaptations to different cell types within WAT

Adipose tissue is a heterogeneous tissue, including adipocytes and the stromal vascular fraction, which comprises pre-adipocytes, mesenchymal cells and immune cells among others (214, 215). Studies have found that exercise-induced modifications to adipose tissue also mediate distinct changes to the various cells residing within adipose tissue which contribute to WAT's endocrine function. One recent study using a mouse model highlighted a unique role of exercise modulated molecular shifts to mesenchymal stem cells in obesity (82). Specifically, 6 weeks of HFD resulted in an increase in extracellular matrix (ECM) remodeling genes in mesenchymal stem cells of WAT with implications in fibrogenesis and inflammatory roles in both humans and rodents (82). Four weeks of exercise attenuated the increased expression of ECM-related genes (82). HFD-induced obesity also downregulated circadian rhythm genes associated with insulin sensitivity and adipogenesis in mesenchymal stem cells, but exercise reversed this effect and increased expression of these genes (82).

Another distinct fining is the identification of oncostatin-M, an exercise-induced adipokine. A recent study showed that the cytokine oncostatin-M was increased in the scWAT transcriptome of patients who were normoglycemic or had T2D after an acute bout of exercise (86). Further investigations showed that oncostatin-M was predominantly produced by the immune cell fraction within scWAT and in vitro treatment of human adipocytes with oncostatin-M results in enhanced MAPK signaling and lipolysis (86). After a 3-hour recovery period, the oncostatin-M receptor gene was increased in skeletal muscle cells, hinting at the possible crosstalk of adipose tissue and muscle via immune cell mediated oncostatin-M response to exercise (86, 216). These data emphasize the role of exercise to mediate potential beneficial effects to specific cell types within WAT which positively contribute to the endocrine function of WAT.

###### Are exercise-induced adaptations to scWAT required for the beneficial effects of exercise?

Given the importance of exercise-induced adaptations to WAT, another study investigated the beneficial effects of exercise in the absence of scWAT by having mice undergo an 11-day exercise protocol after removal of scWAT (135). Surprisingly, scWAT removal had minimal effects on improved glucose and insulin homeostasis in exercised mice, with no compensatory changes observed in other metabolic tissues such as skeletal muscle. This finding provides a unique perspective, as most rodent studies indicate that exercise-induced adaptations to scWAT contribute to improved metabolic health, while these findings suggest that the various exercise-induced adaptations to scWAT and its regulation of glucose and insulin homeostasis are not linked but can occur independently of one another (135).

Together these data indicate that exercise mediates favorable outcomes on the various functions of WAT. Importantly, modulation of WAT's mitochondrial and endocrine activity in both humans and rodents and rodent-specific thermogenic plasticity highlights the potential of WAT to improve metabolic health as a response to exercise, which can be used to combat obesity and obesity-related diseases.

#### Exercise and brown adipose tissue adaptations in obesity

In rodents, brown adipose tissue (BAT) can be found in several regions, including the interscapular, mediastinal, perirenal, axillary, and cervical areas (217). In humans, BAT is predominantly found in the cervical, supraclavicular, axillary, and paravertebral regions (218, 219). BAT has emerged as a potential therapeutic target to combat obesity and cardiometabolic diseases, due to its inverse correlation with the occurrence of T2D and CVD in humans (220). However, the precise underlying mechanisms of how BAT is associated with combating obesity are unknown (218, 221). Alterations to BAT's mitochondrial and thermogenic functions and endocrine activity in obesity, and a potential role for exercise, will be discussed below (Fig. 2B, Table 1).

##### Mitochondrial and thermogenic function of BAT

BAT's thermogenic capacity closely relies on its mitochondrial activity, mainly due to the presence of UCP1 in the mitochondria. Impaired BAT mitochondrial activity has been reported in animals with obesity (222-224). In rodents, HFD induces obesity and hyperglycemia and elevates mitochondrial ROS generation which coincides with increased inflammation in BAT (223). A recent study with mice on HFD for 8 weeks revealed that a BAT-specific deficiency of thioredoxin-2 (TRX2), a mitochondrial redox protein, disrupts mitochondrial function by specifically enhancing the generation of mitochondrial ROS and results in the cytosolic release of mtDNA (225). These mitochondrial aberrations result in the activation of an immune response triggering the cyclic GMP–AMP synthase (cGAS)–stimulator of interferon genes (STING) pathway and NOD-like receptor protein-3 (NLRP3) inflammasome pathways.

Exercise in murine models has shown favorable outcomes in obesity by affecting mitochondrial activity and thermogenesis (67, 90). Studies have shown that 4 to 8 weeks of aerobic exercise (swimming or treadmill) in HFD-induced obese mice increased BAT mass, expression of thermogenic genes, and expression of markers associated with glucose and lipid metabolism (87, 88, 90), and 12 months of treadmill training preserved expression of thermogenic genes in BAT in obese aged mice (89). However, several other studies have shown that exercise training either did not affect BAT mass or UCP1 expression (136, 226, 227) or did not increase Ucp1 protein expression and reduced basal oxygen consumption rates (67). These discrepant data are of interest, as it is not clear why exercise, which is a thermogenic activity, would require an increase in the thermogenic activity of BAT.

With regard to the role of BAT in humans, a recent retrospective analysis of 52 487 patients reported that individuals with the presence of BAT detected via ^18^F-fluorodeoxyglucose positron emission tomography–computed tomography scans (FDG-PET CT) scans had a lower odds of T2D and lesser association with cardiometabolic diseases (220). These results were amplified in individuals with obesity, indicating a role for BAT to attenuate obesity-associated diseases (220). In another human study, there was no difference in BAT volume or activity among lean subjects and subjects with obesity; however, there was a strong inverse correlation between BAT volume, cold-induced thermogenesis, FDG uptake, and visceral adipose tissue (228). Other studies found that metabolically healthy overweight or individuals with obesity had a higher presence of BAT when compared to their metabolically unhealthy counterparts, and individuals with obesity and active BAT had lower visceral fat mass than those without detectable BAT activity (229, 230), highlighting a potential role for BAT to attenuate obesity-associated outcomes.

Studies investigating the effects of exercise on BAT in humans have shown a minimal effect on the thermogenic role of BAT. A comparative study in male subjects showed that 2 hours of cold exposure resulted in significantly lower BAT activity measured by FDG-PET CT scans in endurance athletes when compared to sedentary individuals (231), indicating that exercise training decreased BAT activity. Another study revealed that exercise training reduces insulin-stimulated glucose uptake in BAT in individuals with detectable BAT activity (92). A recent human trial, ACTIBATE, reported that 24-weeks of endurance and resistance training did not affect glucose uptake in BAT or BAT mass, implying that BAT's ability to take up glucose is not affected by exercise (93). The effect of exercise in humans has mostly been investigated in healthy individuals and it is unclear if exercise would induce similar effects in BAT in people with obesity. It is also important to note that the standard measurement technique to measure BAT activity is FDG-PET CT, which solely relies on BAT's ability to take up glucose, using an indirect substrate uptake mechanism to indicate activity. Alternative methods, including infrared thermography and near-infrared time-resolved spectroscopy, have also reported reliable assessment of human BAT (232, 233); T2 mapping, which uses magnetic resonance imaging to measure fat T2 relaxation time, based on BAT having higher water compared to WAT without requiring cold exposure to detect BAT (234, 235) is another alternative method. However, the use of these alternative techniques has not been optimized to determine potential effects of exercise on BAT.

##### Endocrine activity of BAT

Although these studies emphasize that exercise does not increase BAT mass or the ability of BAT to take up glucose, some studies have shown that exercise can possibly alter BAT's endocrine activity (91, 236). In fact, in the previously described ACTIBATE study, endocrine factors from BAT were not measured. Recent studies have identified several factors released from BAT in response to exercise (237); here we will discuss 2 of these batokines, including 12,13-dihydroxy-9Z-octadecenoic acid (12,13-diHOME) and fibroblast growth factor 21 (FGF21) in the context of exercise and obesity.

###### 12,13-diHOME

12,13-Dihydroxy-9Z-octadecenoic acid (12,13-diHOME), is a lipokine released from BAT in response to acute and chronic cold and exercise in humans and is negatively correlated with BMI, adiposity, circulating triglycerides, and insulin sensitivity, and positively correlated with VO_2_ peak (91, 238).

In rodents, 12,13-diHOME is increased in response to both acute and chronic exercise and cold exposure. When mice underwent surgical removal of the interscapular BAT and then were subjected to an acute exercise protocol, there was no elevation in 12,13-diHOME levels, confirming that the exercise-induced increase in 12,13-diHOME was BAT specific (91). Acute treatment of 12,13-diHOME results in increased fatty acid uptake into skeletal muscle both in vivo (Luc ActaCre mice) and in myotubes in vitro (C2C12 cells) and brown adipose tissue, and the heart and increased cardiac function (91, 238, 239), and sustained overexpression of 12,13-diHOME attenuated BW gain and preserved cardiac function in mice fed a high-fat diet (HFD) (239). These data highlight an important endocrine role for BAT in response to exercise.

###### FGF21

FGF21, a hormone primarily released by the liver, plays a crucial role in glucose and lipid metabolism (240, 241). It is released from WAT and BAT in response to cold stimuli and β-adrenergic pathway stimulation in rodents (242-244). Studies have shown that BAT transplantation in mice leads to a 5-fold increase in serum FGF21 concentrations and 2-fold increase in FGF21 protein expression in endogenous BAT which coincided with improved glucose tolerance and increased insulin sensitivity (245, 246).

In humans, studies have shown that neonates have a significant expression of both FGF21 and UCP1 in BAT and mild cold exposure increases circulating FGF21 levels indicating the possible link between FGF21 and BAT related thermogenesis in humans (247, 248).

Acute and chronic exercise increases circulating FGF21 levels in humans (236, 249) and rodents (249), but the direct effects of exercise on FGF21 expression in and release from BAT in response to exercise have not been established. Investigating the effect of BAT to mediate FGF21 in response to exercise in the presence of obesity could potentially reveal new roles for BAT to mediate FGF21. Collectively, these findings suggest that exercise plays a distinctive role in enhancing the endocrine function of BAT while minimally affecting glucose uptake into BAT in human subjects.

#### Obesity driven alterations to the liver

The liver is an essential regulator of whole-body metabolic homeostasis via its role in lipid and glucose metabolism (250-252). Under nonobese conditions, fatty acid storage in the form of triglycerides and fatty acid oxidation form a tightly regulated balance which results in less than 5% of triglyceride levels in the liver (250). Under obese conditions, mechanisms underlying adipose tissue and liver crosstalk are altered. For example, circulating fatty acid levels are increased due to adipose tissue dysfunction, resulting in ectopic storage in the liver. This improper storage contributes to the downregulation of hepatic mitochondrial and cellular functionality, yielding an increase in oxidative stress (253-256). This increase in circulating fatty acid is often coupled with low-grade systemic inflammation which promotes the activation and infiltration of hepatic immune cells and consequently inflammation and fibrogenesis (253-256). These cellular and molecular abnormalities have prompted the generation of a large body of studies which have shown that obesity can contribute to the development and progression of metabolic dysfunction–associated fatty liver disease (MAFLD) and MASH (257-261).

Exercise has emerged as one of the key lifestyle modifications recommended to patients with obesity concurrently diagnosed with MAFLD/MASH (31, 46, 52, 262-265). The effects of exercise to potentially ameliorate obesity-related MAFLD and MASH will be discussed below.

##### Exercise-induced modulations to the liver in obesity

Numerous studies have reported altered glucose and lipid metabolism in MASH with significant effects on pathways involved in glycolysis, gluconeogenesis, and fatty acid oxidation. Animal models of diet-induced obesity have shown an upregulation of enzymes involved in glycolysis, like hexokinase 2, phosphofructokinase muscle isoform, and pyruvate kinase muscle isoform (266) and enhanced hepatic gluconeogenesis (267-269). Modifications in lipid metabolism also contribute to MASH progression, including increased de novo lipogenesis, lipid uptake, and fatty acid oxidation. Fatty acid oxidation–related genes such as PPARα, PGC1α, and CPT1α and fatty acid translocase receptors CD36 and FAT binding protein 1 (FABP1) are significantly upregulated in animal fatty liver models indicating the increased uptake and oxidation of fatty acids which consequently promote the disruption of hepatic insulin sensitivity (270-274).

Exercise is highly effective in mitigating MASH and MAFLD. Studies show that various forms of exercise enhance insulin signaling, improve glucose tolerance, and reduce liver steatosis in animal models (264, 275-279). For instance, HIIT improves glucose tolerance and decreases markers of hepatic lipogenesis such as PPARγ, diacylglycerol O-acyltransferase 1 (Dgat1), acetyl-CoA carboxylase alpha (Acaca), and acetyl-coenzyme A carboxylase beta (Acacb) (264). Four weeks of exercise also decreased gluconeogenesis enzymes such as fructose-1,6-bisphosphatase 1, alongside increased Ser473-phosphorylation, suggesting the activation of the PKB/Akt insulin signaling pathway (278). Twelve weeks of strength training in DIO rats also correlated with reduced hepatic fatty acid storage and fatty acid uptake receptor CD36 expression, alongside lipogenesis marker sterol regulatory element-binding transcription factor 1 (SREBP1) expression and a 12-week swimming protocol in HFD-fed mice decreased of FABP1 (276, 279). Exercise also increased activation of AMPK, an important factor in fatty acid oxidation in the liver (262). This potentially integral role of fatty acid oxidation re-establishment was corroborated in a separate study in which mice were exposed to either MIT or HIIT for 8 weeks (14). Both training regimens resulted in increased circulating levels of adiponectin, and an increase in hepatic adiponectin-mediated fatty acid oxidation markers such as sirtuin 1 (SIRT1), PPARα, CPT1a, cytochrome P450 Family 2 Subfamily E Member 1 (Cyp2e1), and insulin receptor substrate 2 (Irs2) in conjunction with significantly lower hepatic glycogen and lower hepatic mRNA levels of SREBP1c, Fas Cell Surface Death Receptor (FAS), CD36, and lipin1 (14). Additionally, exercised mice had increased levels of hepatic pAMPK/AMPK ratio and reduced glycogen content (14).These preclinical studies demonstrate the potential of exercise in reestablishing proper hepatic fatty acid oxidation functionality through crosstalk between AT and the liver which can play a mitigating role in the progression of liver disease.

In human studies, patients with steatohepatitis have increased hepatic glucose phosphorylation and those with elevated intrahepatic triglycerides have higher endogenous glucose production and very low-density lipoprotein triglycerides from hepatic de novo lipogenesis (280-283). MAFLD patients also have increased lipogenesis and liver X receptor (LXRa) levels, which promote lipogenesis via SREBP1C activation (284-286). A study that metabolically profiled tissue-specific insulin resistance in individuals who were overweight or have obesity revealed high levels of circulating branched chain amino acids such as valine and isoleucine, triglycerides, lactate and reduced glycine levels which correlated with liver specific insulin resistance (287).

Exercise benefits individuals with MASH by reducing intrahepatic lipid content, improving insulin sensitivity, and maintaining glucose homeostasis (288-291). Two weeks of resistance training, high-intensity interval aerobic training, and moderate intensity continuous aerobic training decreased hepatic fat content, liver stiffness, and inflammatory markers like leptin and ferritin (288). Additionally, 12 weeks of a combined aerobic and resistance training program resulted in decreased intrahepatic lipid content and improved peripheral insulin sensitivity by 23% for individuals with MAFLD (292). Human studies have also yielded consistent results demonstrating the importance of aerobic exercise training in reducing expression of inflammatory markers. Specifically, both circulating TNF-α and IL-6 were decreased, alongside a reduced expression of oxidative stress markers (31, 293, 294). Additionally, a post hoc analysis of liver biopsies from the NASHFit trial reported that a 20-week moderate intensity exercise routine correlated with reduced liver fat, as well as reduced levels of FGF21 levels (295). FGF21 has been implicated in MASH progression, due to disrupted lipid oxidation pathways (295). MASH and MAFLD has been associated with a FGF21-resistant state rendering FGF21 and its analogues as effective therapeutic options for liver disease (296-299). Interestingly, a simple resistance training protocol consisting of pushups and squats for 12 weeks or walking for 200 minutes per week for a year corresponded with a decrease in hepatic steatosis, regression of hepatic fibrosis, and lower FGF21 levels (15, 265).

Taken together, both animal and human data point toward the metabolic remodeling capacity that exercise can induce in obese conditions, as well as demonstrate a potential hepato-protective effect against the development of further liver disease.

#### Obesity-induced modifications to the skeletal muscle

Skeletal muscle is one of the most metabolically active organs in the body, responsible for up to 80% of insulin-stimulated glucose uptake and disposal under nonobese conditions (300, 301). However, in cases of obesity, numerous muscular metabolic pathways can be adversely altered, reducing insulin sensitivity and impairing function of insulin signaling receptors and key glucose transporters (16, 301-303). Obesity disrupts lipid metabolism pathways in muscle, resulting in increased lipid accumulation and mitochondrial dysfunction preceding incomplete oxidation of fatty acids (16, 304).

Similar to other metabolically relevant organs, exercise training improves skeletal muscle glucose and lipid metabolism and mitochondrial function, thus attenuating the negative impacts of obesity (16, 305). Exercise-induced adaptations to skeletal muscle as a potential method to improve metabolic regulation in obesity will be discussed below.

##### Exercise-mediated skeletal muscle adaptations in obesity

Skeletal muscle is a vital organ for maintaining metabolic balance through its significant contribution to energy expenditure, insulin response, and ability to adapt to the body's metabolic demand, via its mitochondrial content and oxidative capacity (306-308). However, under obese conditions, skeletal muscle exhibits reduced insulin-mediated glucose uptake, impaired oxidative metabolism, and increased lactate production, which is often associated with impaired insulin signaling and glucose metabolism pathways, such as reducing glucose transport, glycogen synthesis, and glucose oxidation (302, 303, 309-312). Additionally, lipid metabolic pathways are also negatively affected, including an enhanced fatty acid transport system, leading to increased fatty acid esterification and higher intramuscular triacylglycerol levels (313, 314).

HFD in animal models results in muscular lipid accumulation, insulin resistance, and a reduction of mitochondrial biogenesis markers such as AMPK and PGC1a, contributing to metabolic dysfunction (315-318). Murine studies have shown that aerobic/endurance exercise enhances insulin signaling via upregulation of AKT, as well as translocation of the glucose transporter GLUT4 in obese mice (319, 320). AMPK also plays a critical role in insulin-stimulated glucose uptake by skeletal muscle after exercise via the inhibition of the Rab-GTPase–activating protein TBC1D4, which consequently results in the translocation of GLUT4 to the membrane and enhanced glucose uptake (321). A whole-body TBC1D4 knock-in mouse model showed that after an acute bout of treadmill exercise, improvement in whole-body and muscle insulin sensitivity was dampened after exercise (321). These findings are corroborated in a notable human study where skeletal muscle from individuals with *TBC1D4* p.Arg684Ter variant displayed a reduced post-exercise insulin sensitization effect (322). Specifically, individuals with the *TBC1D4* p.Arg684Ter variant had up to 50% of reduced glucose uptake in the skeletal muscle after 1 hour of exercise. Exercise in rodent studies has also shown that moderate intensity endurance training in rats increases oxidative phosphorylation, lipid oxidation, and mitochondrial biogenesis and decreases mitochondrial stress in skeletal muscle (323).

In humans, obesity is associated with metabolic impairments in skeletal muscle, including diminished insulin-induced glucose uptake, reduced oxidative metabolism, and increased lactate production (303, 310). Individuals with obesity have reduced insulin-stimulated phosphorylation of IRS1 and Akt and lipid oxidative capacity and higher levels of intramuscular triacylglycerol (304, 312, 324, 325). Like in rodents, human studies have shown that endurance exercise increases fatty acid oxidation, reduces intramuscular triglyceride accumulation, and inflammation in skeletal muscle (326-328). One study investigating women with obesity revealed that 12 weeks of combined aerobic and resistance training from moderate to vigorous intensity resulted in changes to the skeletal muscle lipid intermediate levels, such as cardiolipin and phosphatidylcholine, which was accompanied by an increase in mitochondrial respiration (329). Other studies using endurance exercises have shown a decrease in intermuscular adipose tissue for older individuals with obesity, and body fat reduction alongside improvements in muscle mitochondrial content for diet-resistant women with obesity (330, 331). Additionally, in the context of obesity, exercise-induced myokines, which include cytokines, small proteins, and peptides released from the skeletal muscle, also undergo alterations (332). Some myokines affected by obesity and exercise include IL-6 which plays an anti-inflammatory role alongside improving insulin-stimulated glucose uptake and glucose transporter GLUT 4 translocation in skeletal muscle (333, 334), metrnl which has been linked to worsening glucose tolerance and plays a role in thermogenic and energy expenditure pathways (335, 336) and irisin which is associated with insulin resistance and has been implicated in the browning of white fat among its musculoskeletal roles (337-339). Detailed mechanisms of additional myokines including IL-6, metrnl, and irisin and their role in mediating metabolic diseases are thoroughly reviewed elsewhere (332, 340).

Overall, these studies provide compelling evidence that exercise is an effective and powerful tool to alleviate the detrimental effects of obesity and improve overall health. However, escalating levels of obesity and associated diseases pose a significant global health challenge and necessitate the advancement of additional mechanisms that could be used as a combinatorial approach with exercise.

### Pharmacotherapeutics, Bariatric Surgery, and Exercise: Potential for Combined Therapies?

Alternative methods, including incretin therapies and bariatric surgery, have presented promising results in weight reduction with improved cardiovascular outcomes (17, 18, 341-343). These promising outcomes may aid in progressing health benefits for individuals that have less impactful effects from conventional methods to reduce fat mass and improve metabolic health. Moreover, with the advantageous effect of exercise on obesity, combinations of these methods and exercise could likely exploit effectual mechanisms that would aid in enhanced treatment of larger groups of individuals with obesity.

Reviewing current findings on the impacts of incretin therapy and bariatric surgery in combination with exercise in humans provides a holistic outlook on the potential of synergistic approaches to combat obesity (Table 2).

**Table 2.** Recent findings on outcomes of combination of incretin therapy, bariatric surgery, and exercise in humans

Combinatorial therapies in human studies	Weight loss procedure	Interventions	Outcomes
Incretin therapy and exercise	Liraglutide	1 year of liraglutide at 3.0 mg/day and moderate to vigorous intensity exercise 4 times a week (344)	Abdominal fat percentage reduced by 6.1% Metabolic syndrome severity z-score decreased by 0.48
		1 year of liraglutide at 3.0 mg/day and moderate to vigorous exercise 4 times a week (21)	Weight loss of 9.5 kg Body fat reduction by 3.9% Improvements in insulin sensitivity and glycated hemoglobin
		1 year of liraglutide at 3.0 mg/day and moderate to vigorous intensity exercise 4 times a week (345)	6.88 kg of weight loss change Unchanged bone mineral density at hip and lumbar spine
		1 year follow up after termination of intervention from same cohort that previously received 1 year of liraglutide at 3.0 mg/day and moderate to vigorous intensity exercise 4 times a week (346)	Reduced body weight of approximately 5.1 kg Reduced body fat percentage of 2.3% Weight regain of 2.5 kg
	Semaglutide	20 weeks of 0.5 mg or 1.0 mg of semaglutide weekly then combined with aerobic exercise (average heart rate reserve of 75% of maximum) 3 times per week for 12 weeks in patients with T2D (347)	Improved body fat percentage Improved glycemic control Improved pancreatic beta cell insulin secretion
	Tirzepatide	6 weeks of 2.5 mg or 5.0 mg of tirzepatide weekly and 3 sessions per week of resistance and aerobic exercises (348)	Reduced body weight, waist circumference, fat mass, and waist to hip ratio Exercise did not have an additive effect on fasting blood glucose and triglyceride levels
Bariatric surgery and exercise	Presurgery	Aerobic dance-based exercise for 60 minutes, 2 days a week for 8 weeks. Analysis after 8 weeks of intervention and 5 months post SG (349)	Improved functional capacity Improved muscle strength and endurance Improved physical activity Improved fatigue scores These results were seen both at 8 weeks post intervention and 5 months postsurgery
		12 weeks of endurance and strength training. 3 sessions per week for 80 minutes and monthly aqua gym (350)	Improved 6-minute walking test Increased half-squats Increased arm curl repetitions Improved social interaction score
		1 year postsurgery RYBG or SG evaluation of presurgery exercise intervention mentioned previously (350, 351)	Increased physical activity Increased 6-minute walking test Increased half-squat test Decreased BMI
		Aerobic and stretching exercises, 25 minutes each, 2 sessions weekly in addition to cognitive-behavioral therapy (CBT), once a week for 4 months (352)	Reduced body weight for the exercise and exercise + CBT groups Reduced BMI for the exercise group and exercise + CBT group Improved functional capacity and cardiometabolic parameters such as blood pressure for both exercise and exercise + CBT groups
		Aerobic (including HIIT) and resistance training, 2 sessions per week for 6 months (353)	Reduced BMI Reduced fat mass Improved blood pressure
	Postsurgery	Resistance training for 1 hour, 3 times a week for 18 weeks post RYGB in addition to supplemental whey protein dose of 48 grams/day (354)	Increased lower-limb muscle strength
		5-year postsurgery follow up of previously mentioned intervention (20, 354)	Increased physical activity Lower weight regain
		60-min group exercise classes with functional strength, flexibility, and aerobic activities, 2 times per week for 6 months and at least 3 days per week of self-directed exercise post RYG, SG, and GB (355)	Increased aerobic fitness after 6 months of intervention that lasted an additional 6 months with maintenance
		12 weeks of aerobic and strength training, 3 times per week post RYGB and SG (356)	Reduced weight Reduced percent body fat Reduced fat mass Increased change in 12-minute walk test
		Resistance training for 12 weeks, 60-80 minutes, 3 times a week post RYGB (357)	Improved muscle strength and quality including less press strength, leg extension strength, and leg press quality
		Aerobic and resistance training for 60 minutes, 3 times a week for 12 weeks post RYGB, SG, and GB (358)	Decreased fat mass Improved physical function
		Aerobic and resistance exercise up to 74 minutes, for 5 months separated into 5 blocks for every 4 weeks post SG (359)	Reduced fat mass Reduced blood glycemic levels Reduced cholesterol levels
		Aerobic exercise for 120 minutes, 3 to 5 times per week for 6 months post RYGB (360)	Reduced fat mass Reduced abdominal adipose tissue Maintenance of skeletal muscle mass

Abbreviations: BMI, body mass index; GB, gastric banding; HIIT, high-intensity interval training; RYGB, Roux-en-Y gastric bypass; SG, sleeve gastrectomy; T2D, type 2 diabetes.

#### Incretin therapy and exercise

Incretins are a group of hormones that are released by the gastrointestinal tract in response to nutrient uptake and have physiological actions on multiple organs. Specifically, incretins endogenously function to stimulate glucose-stimulated insulin secretion by pancreatic β-cells and simultaneous reduction in the secretion of glucagon and slowing of gastric emptying, which promotes satiety and reduces appetite (341, 342). Multiple studies have linked the secretion of glucagon-like peptide 1 (GLP-1) to obesity (361-365). For instance, individuals with obesity have lower plasma GLP-1 levels compared to lean controls following a solid meal test, which coincides with higher gastric emptying in the obese subjects (361, 365). Furthermore, the large ADDITION-PRO study, which included 1462 participants, found that individuals classified as obese or overweight showed a reduction of up to 20% in plasma GLP-1 levels following an oral glucose test (363). Notably, BMI and WC were negatively correlated with GLP-1 levels (363).

Several incretin therapies have been studied as therapeutics for obesity. Among incretins, GLP-1 (and specifically GLP-1 receptor agonists [GLP-1 RAs]) have gained the most popularity in treating T2D and obesity, with additional cardiovascular benefits (18, 341, 342). GLP-1 RAs are altered versions of GLP-1 which mimic its biological activity, conferring the benefits of lowering blood glucose levels with an extended half-life and avoiding severe hypoglycemic states (366, 367). Currently the FDA has approved 3 GLP-1 RA s for the treatment of obesity: liraglutide, semaglutide, and tirzepatide (the latter is a GLP-1/glucose-dependent insulinotropic polypeptide [GIP] dual agonist). Studies on liraglutide have demonstrated that daily treatment when combined with lifestyle interventions, including dietary deficits and physical activity, for 56 weeks can result in significant weight loss with a reduction of up to 25.2% (368-370). Forty weeks of combined liraglutide treatment and physical activity also affects the visceral adiposity of individuals, leading to a reduction of vWAT by 12.49% (371). Similarly, semaglutide treatment for 68 weeks, alongside lifestyle modifications, led to significant reductions in BW, BMI, vWAT, and cardiometabolic risk factors such as lipid levels and blood pressure (18, 372, 373). Tirzepatide studies showed significant weight loss as well, with reductions up to 25.3% and notable improvements in cardiometabolic parameters like WC, fasting insulin, and lipid levels across various doses (374-377).

Although GLP-1 RAs mediate favorable effects in the setting of obesity, the benefits of GLP1-RAs are accompanied by conflicting results about bone health, particularly the risk of bone fractures and reduced bone mass density (378-380). Weight regain after the termination of GLP-1 RAs treatments is also a concern for current therapeutic strategies (381, 382). Less is known about the combined effects of exercise and semaglutide or tirzepatide. One study showed that a combination of semaglutide and aerobic exercise for 12 weeks in T2D individuals with prior semgalutide use for 20 weeks directly enhances insulin secretion, body composition parameters such as body fat, and glycemic control (347). A 68-week study demonstrated that weekly semaglutide administration in combination with 150 minutes of weekly physical activity resulted in 14.9% of BW reduction when compared to the control groups (18). However, once treatment was terminated, participants regained approximately two-thirds of BW lost in a year and the benefits to cardiometabolic risks were reversed (18, 382). In the case of tirzepatide treatment, a 6-week study combining tirzepatide and aerobic and resistance training showed no additive effects of exercise to fasting blood glucose and triglyceride levels (348).

Studies investigating the combined effects of liraglutide and moderate to vigorous intensity exercise reported improvements in multiple metabolic health parameters, such as insulin sensitivity, hemoglobin glycation levels, and reduced abdominal obesity and BFP (21, 344), while a year-long regimen helped preserve bone mass density in the hip and lumbar spine (345). This suggests that the combination of liraglutide and exercise not only provides metabolic and inflammation related benefits but also supports bone health. Interestingly, Jensen et al studied the long-term benefits of daily liraglutide in combination with vigorous exercise 1 year after the termination of interventions (346) and found that participants who had received a combination of liraglutide and exercise had maintained weight loss up to 10% of initial BW and the same group had a weight regain of only 2.5 kg 1 year after termination of treatment, and increased physical activity when compared to the control groups, suggesting that vigorous exercise could potentially prolong beneficial effects of GLP1-RAs directly or indirectly through encouraging healthy physical activity habits (346).

These recent findings imply that any form of physical activity strengthens the effects of GLP1-RAs. Moderate to vigorous exercise amplify the impacts of GLP1-RAs on weight loss and overall metabolic health. Extensive studies would be required to identify the exact synergistic mechanisms of exercise and GLP1-RAs in curbing the adverse effects reported with GLP-1 RAs therapy alone.

#### Bariatric surgery and exercise

Bariatric surgeries are a set of stomach or intestinal procedures aiming to achieve long-term weight loss in cases of severe obesity, with results of weight loss up to 25% at 10 years after intervention (17, 343, 383, 384). Some of the most common procedures include Roux-en-Y gastric bypass (RYGB) and the sleeve gastrectomy (SG) (17). Bariatric surgery enhances transcriptional signatures for mitochondrial oxidative phosphorylation in scWAT (385, 386) and can alter circulating factors such as IL-27 (387). With the drastic weight loss after bariatric surgery comes the long-term adverse effects of decreased muscle strength, weight regain, and protein and micronutrient deficiencies (20, 381, 388, 389). Notably, studies have demonstrated that exercise training, specifically aerobic and resistance training, improve clinical outcomes such as greater fat loss, longer 6-minute walking distance, lower systolic blood pressure, and increased muscle strength up to 1 year after bariatric surgery (Table 2) (20, 354-360). A study conducted with 76 female participants after RYGB analyzed a combination of resistance training and protein supplementation following bariatric surgery (354). The results showed that 6 months postsurgery, participants who received additional whey protein intake of 48 grams per day in addition to 3 weekly resistance training sessions had increased lower-limb muscle strength when compared to control groups (354). A 5-year follow up of this study showed that even though muscle strength decreased over time in patients with protein supplementation and exercise, there was an increase in physical activity levels, which positively correlated with lower weight regain postsurgery (20).

Preoperative exercise interventions are beneficial for patients prior to bariatric surgery (Table 2) (349-353). A recent study reported that a presurgery aerobic dance-based exercise program for 60 minutes, twice a week for 8 weeks results in increased muscle strength and endurance, physical activity levels, functional capacity, and quality of life when compared to the group that only received physical activity counseling and these effects were sustained up to 5 months after surgery (349). Additionally, longer durations of presurgery exercise including 6 months of aerobic and resistance training showed improvements in BMI, BFP, and blood pressure (353).

These studies collectively highlight the importance of exercise in achieving long-term beneficial outcomes of bariatric surgery, emphasizing exercise as a critical component in obesity management. Furthermore, it is crucial to recognize that many findings, as shown in Table 2, demonstrate that the positive effects of exercise—such as enhanced insulin sensitivity, increased muscle strength, and reduced fatigue—are independent of weight loss. This underscores the fact that the advantageous adaptations of exercise in obese individuals are not solely driven by changes in BW.

### Can Exercise Override the Genetic Causes of Obesity?

The causes of obesity are multifaceted and various genetic and environmental factors contribute to disease development (390-393). Several environmental factors are modifiable, including things like diet and sedentary lifestyle, and there are multiple studies that discuss how exercise can combat these environmental factors (394-396).While lifestyle factors are important contributors to the pathogenesis of obesity, genetic factors also play a significant role. Monogenic or polygenic disorders to critical genes or regulatory processes can result in the development of nonsyndromic obesity, which causes early-onset obesity (397, 398). Nonsyndromic obesity is primarily associated with genetic mutations to factors involved in the leptin-melanocortin pathway and presents as a disruption to energy homeostasis and its monogenic form affects approximately 5% of the population with early-onset obesity (398-401). Aberrations at the gene, chromatin, and RNA-associated post-transcriptional modification levels can contribute to the development of nonsyndromic obesity, highlighting the genomic complexity of the disease. While the genomic impacts on disease are most likely irreversible, exercise may be a powerful tool to mitigate the extent to which these factors can contribute to disease onset and progression.

#### Nonsyndromic obesity–associated genes and exercise

Multiple genes have been identified by emerging research as being potential contributors to the development of nonsyndromic obesity. Genetic mutations resulting in variants of LepR, such as K109R, Q223R, and K656N, have an increased association with obesity (402-406). Chavez et al followed a family over 3 generations and found that early-onset obesity and delayed puberty observed within individuals of the family were associated with mutations to LepR (407). While the role of exercise in mediating the effects of this type of genetic aberration are unknown, physical activity has been shown to have beneficial effects on individuals with a greater likelihood for higher BMIs by attenuating genetic effects on obesity including leptin and LepR single nucleotide polymorphisms (SNPs) (408, 409). Thus, it is possible that exercise could be beneficial to ameliorate some of the consequences of genetic obesity.

Another nonsyndromic obesity–related gene is the fat mass and obesity associated gene (FTO), which encodes the FTO protein that demethylates N^6^-methyladenosine (m^6^A) and is essential for adipogenesis (410-412). Genome-wide association studies found that single nucleotide polymorphisms in the FTO gene were associated with obesity parameters such as BMI; however, the proportion of population affected by alterations to the FTO genes greatly vary on the population being studied as population frequencies have been reported up to 46% in Western and Central Europeans and up to 29% in Asians (413-418). The well-investigated rs9939609 polymorphism was associated with increased BW and BMI and was shown to influence appetite and fat oxidation during exercise (419-421). Interestingly, physical activity reduces the association between *FTO* rs9939609 and the odds of obesity (422). The beneficial effects of exercise were additionally observed in individuals with the *FTO* rs1421085 variant as when individuals with this risk variant regularly exercised, a lesser weight gain and an increase in BMI was observed (423). Together, these studies demonstrate that diverse genes and their associated mutations can increase the risk of obesity predisposition. However, this risk can partially be mitigated by exercise, demonstrating its importance as a tool for destratification of altered gene activity and disease onset.

#### Nonsyndromic obesity–associated regulatory mechanisms and exercise

Regulation of gene activity via chromatin accessibility is an extensively established field of research often associated with various diseases. DNA methylation is one of the broadly studied epigenetic mechanisms that has been associated with nonsyndromic obesity regulation (424-426). Notably, numerous cytosine-phosphate-guanine (CpG) sites within obesity-associated genes have enriched DNA methylation (424, 427-429). Specifically, studies have found that DNA methylation is associated with alterations to BMI and WC (427-430). Interestingly, a study analyzing blood samples from subjects that conducted an 18-month low-fat or low-carbohydrate diet with and without exercise showed that CpG sites for genes associated with obesity were negatively correlated with changes to BW after the diet and exercise intervention (431).

In addition to chromatin modulations dictating gene accessibility, gene product modifications also play a noteworthy role in nonsyndromic obesity onset. MiRNAs are noncoding RNAs which regulate post-transcriptional modifications to the genome and have been reported to have effects on adipogenesis and adipose tissue inflammation response (432, 433). Various miRNAs have been associated with obesity (BMI levels) and body fat distribution in children and adults (434, 435).

Exercise alters miRNA profile in individuals with obesity (436). Specifically, a 3-month long physical activity intervention resulted in a decrease in circulating miR-146a-5p with a strong correlation with WC and inflammatory cytokine IL-8 (437). Together, these studies demonstrate the crucial role of regulatory elements at both the DNA and RNA levels in predisposing individuals to obesity, as well as the role of exercise in mitigating these epigenetic and post-transcriptional modification factors.

Emerging data demonstrates that exercise can potentially affect genetic obesity, but there are significant limitations. For instance, it is unlikely that exercise could completely change gene expression. It is more likely that exercise can modify epigenetic alterations that impact gene expression related to whole-body metabolic function which can attenuate or circumvent the negative effects conferred by genetic alterations. This emphasizes the concept that obesity is a complex disease induced by numerous genomic and environmental factors, and that no single treatment option may be powerful enough to truly overcome the disease alone.

## Future Directions

Exercise induces beneficial metabolic changes to WAT, BAT, liver, and skeletal muscle in both humans and rodents, mitigating the adverse effects of obesity. While distinct mechanisms within the two species exist, such as exercise triggering a beiging response in WAT of rodents, it is important to acknowledge that other functions, such as enhanced endocrine activity and mitochondrial activity, play an important role in exercise-induced adaptations in obesity. Multiple factors determine the effectiveness of exercise-induced adaptations, including sex, genetic aberrations, duration, modality, temperature, and metabolic health status. When discussing exercise and its notable benefits, an important consideration is Pontzer's constrained energy expenditure hypothesis, which suggests that physical activity minimally affects daily caloric burn, with nonexercised activity thermogenesis (NEAT) and dietary patterns playing key roles (438). NEAT decreases with excessive exercise unless dietary compensation occurs (439) and greater efficiency in physical activity may further reduce total energy expenditure (440-442). These insights highlight the need for comprehensive strategies that address behavioral and metabolic complexities. Current ongoing studies investigating a possible combinatorial therapeutic strategy with pharmacotherapeutics, bariatric surgery, and exercise to curb the adverse effect of obesity report promising results in minimizing drawbacks of extreme weight loss strategies, reinforcing exercise's potential as a compelling therapeutic tool.

## Conclusion

Obesity is a complex, multifactorial disease that encompasses metabolic changes to associated organs such as adipose tissue, liver, and skeletal muscle. A combination of genetic and environmental factors has been shown to play a crucial role in the pathogenesis of obesity, with a lifestyle change including exercise emerging as first-line therapy to treat the disease. With the rapid growth in obesity levels worldwide, the urgency to explore new therapeutic strategies has led to numerous intensive treatment options such as bariatric surgery and incretin therapy. While these studies are promising, factors such as sex differences, age, fitness measurement techniques, accuracy of anthropometric measurements, and their potential contribution to exercise-induced adaptations to combat obesity should be considered to fully elucidate the beneficial effects of exercise to increase efficacy. A continued understanding of how multiple contributing factors in obesity modulate exercise-induced benefits to key organs and metabolic health will potentially provide therapeutically relevant targets to combat obesity.

## Abbreviations

12,13-diHOME: 12,13-dihydroxy-9Z-octadecenoic acid
AdEVs: adipose-derived extracellular vesicles
AMPK: AMP-activated protein kinase
BAT: brown adipose tissue
BFP: body fat percentage
BMI: body mass index
BW: body weight
CPT1: carnitine palmitoyl transferase I
CVD: cardiovascular disease
DIO: diet-induced obesity
EVs: extracellular vesicles
FDG-PET CT: ^18^F-fluorodeoxyglucose positron emission tomography–computed tomography
FGF21: fibroblast growth factor 21
FTO: fat mass and obesity-associated (gene)
GLP-1: glucagon-like peptide 1
GLP-1 RA: glucagon-like peptide 1 receptor agonist
HFD: high-fat diet
HIIT: high-intensity interval training
IL-: interleukin
KYN: kynurenine
KYNA: kynurenic acid
LepR: leptin receptor
MAFLD: metabolic dysfunction–associated fatty liver disease
MASH: metabolic dysfunction–associated steatohepatitis
MICT: moderate intensity continuous training
miRNA: microRNA
MIT: moderate intensity exercise
NRF1: nuclear respiratory factor 1
OXPHOS: oxidative phosphorylation
Pgc1a: peroxisome proliferator-activated receptor-γ coactivator
Prdm16: PR domain containing 16
ROS: reactive oxygen species
RYGB: Roux-en-Y gastric bypass
scWAT: subcutaneous white adipose tissue
SG: sleeve gastrectomy
SREBP1: sterol regulatory element-binding transcription factor 1
T2D: type 2 diabetes
Tfam: mitochondrial transcription factor A
TGF-β2: transforming growth factor beta receptor 2
TNF-α: tumor necrosis factor alpha
Ucp1: uncoupling protein 1
vWAT: visceral white adipose tissue
WAT: white adipose tissue
WC: waist circumference
